# Investigation of the Role of Hydrophobic Amino Acids on the Structure-Activity Relationship in the Antimicrobial Venom Peptide Ponericin L1

**DOI:** 10.1007/s00232-021-00204-y

**Published:** 2021-11-18

**Authors:** Nicholas P. Schifano, Gregory A. Caputo

**Affiliations:** 1 Department of Chemistry & Biochemistry, Rowan University, 201 Mullica Hill Road Glassboro, NJ 08028; 2 Department of Molecular & Cellular Biosciences, Rowan University, 201 Mullica Hill Road Glassboro, NJ 08028

**Keywords:** Venom peptides, antimicrobial peptides, fluorescence, model membranes, antibacterial

## Abstract

Venom mixtures from insects, reptiles, and mollusks have long been a source of bioactive peptides which often have alternative uses as therapeutics. While these molecules act in numerous capacities, there have been many venom components that act on the target cells through membrane disruptive mechanisms. These peptides have long been of interest as potential antimicrobial peptide platforms, but the inherent cytotoxicity of venom peptides often results in poor therapeutic potential. Despite this, efforts are ongoing to identify and characterize venom peptide which exhibit high antimicrobial activity with low cytotoxicity and modify these to further enhance the efficacy while reducing toxicity. One example is ponericin L1 from *Neoponera goeldii* which has been demonstrated to have good antimicrobial activity and low *in vitro* cytotoxicity. The L1 sequence was modified by uniformly replacing the native hydrophobic residues with either Leu, Ile, Phe, Ala, or Val. Spectroscopic and microbiological approaches were employed to investigate how the amino acid sequence changes impacted membrane interaction, secondary structure, and antimicrobial efficacy. The L1 derivatives showed varying degrees of bilayer interaction, in some cases driven by bilayer composition. Several of the variants exhibited enhanced antimicrobial activity compared to the parent strain, while others lost all activity. Interestingly, the variant containing Val lost all antimicrobial activity and ability to interact with bilayers. Taken together the results indicate that peptide secondary structure, amino acid composition, and hydrophobicity all play a role in peptide activity, although this is a delicate balance that can result in non-specific binding or complete loss of activity if specific amino acids are incorporated.

## Graphical Abstract

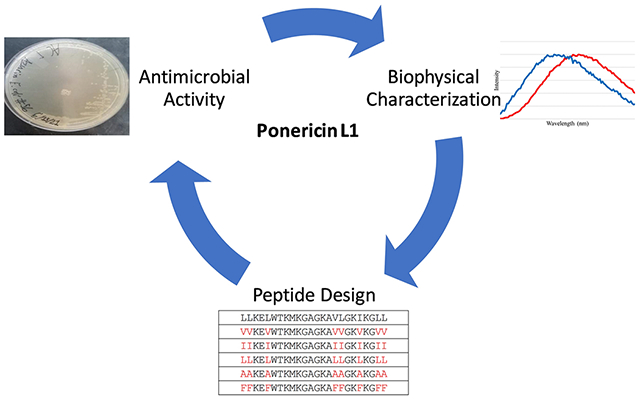

## Introduction

The increasing development of antibiotic resistance across the globe has emerged as a major threat to world health (CDC, 2019; Organization, 2018). There have been numerous and varied approaches to addressing the growing antimicrobial resistance threat including public health and informational efforts, changes in clinical and prescription practices, and the continued development of novel antimicrobial compounds. However, due to the rapid emergence of resistance and the costs attached to the research, development, and approval process for new drugs, traditional small molecule antimicrobials are no longer being developed at a rate that can stem the tide of antimicrobial resistance (Lepore et al., 2019). Instead, numerous novel platforms and delivery mechanisms have been an area of significant research including polymers, phage therapy, coatings and surfaces, bacterial quorum sensing inhibitors, and antimicrobial peptides (AMPs)(Buccini, Cardoso & Franco; Flockton et al., 2019; Foster, Yusa & Kuroda, 2019; Goderecci et al., 2017; Palermo et al., 2019; Schooley et al., 2017).

Antimicrobial peptides are typically short (10-40aa), amphiphilic, cationic peptides that often form helical conformations when bound to bacterial surfaces (Buccini et al.; Makhlynets & Caputo, 2021). AMPs are very promising for therapeutic development due to the high selectivity between bacterial and host cells which these molecules exhibit due to the electrostatic attractions between the cationic peptides and the anionic bacterial cell surface. Additionally, the majority of AMPs are known to act through membrane-disruptive mechanisms, which in turn presents challenges to the development of bacterial resistance since bacteria cannot easily evolve major structural changes to the lipid membrane structure (Buccini et al.; Makhlynets & Caputo, 2021). However, despite the potential, AMPs have faced challenges translating into clinical settings due to poor bioavailability profiles and incompatibility with oral delivery due to host protease degradation (Arenas et al., 2016; Mahlapuu et al., 2016; Mohamed, Abdelkhalek & Seleem, 2016).

One related class of molecules are membrane disrupting venom peptides. While these peptides act similarly to AMPs in causing destabilization of the bilayer membrane, they did not evolve specifically as antimicrobial, but general offensive/defensive venom components. However some of the most well characterized membrane-active venom peptides, melittin from *Apis mellifera* and mastoparan from *Vespula lewisii*, as well as many other venom peptides exhibit the characteristic cationic, amphiphilic amino acid composition and helical propensity found in traditional AMPs (Chrom, Renn & Caputo, 2019; Raghuraman & Chattopadhyay, 2006; Tang et al., 2007). These molecular properties make these venom peptides an additional and promising source for the development of novel antimicrobials (Manniello et al., 2021).

The ponerine ant, *Neoponera goeldii* (previously classified as *Pachycondyla goeldii*), produces a mixture of venom peptides known as ponericins (Orivel et al., 2001). These peptides have been subdivided into three sub-classes based on the amino acid sequences (L, W, and G) and contain a total of 15 independent peptide sequences. When originally isolated, the peptides were demonstrated to have good broad-spectrum antimicrobial activity as well as selective insecticidal action. Among these peptides, the L1 sequence was the most poorly characterized. Subsequently, our group recently demonstrated the importance of the cationic residues in the L1 peptide antimicrobial activity, and that the peptide mechanism of action is likely through disruption of the bacterial membrane (Senetra, Necelis & Caputo, 2020b). Importantly, this work also demonstrated a very low level of hemolytic activity for the L1 peptide and derivatives, a promising first step in the development of these molecules as therapeutics.

In this study, the role of the hydrophobic amino acids in ponericin L1 was investigated by uniformly replacing the naturally occurring hydrophobes with other naturally occurring hydrophobic residues. The hydrophobicity of antimicrobial peptides and peptidomimetics has been shown to play an important role in both the activity and cytotoxicity of these molecules (Edwards et al., 2016; Greco et al., 2020; Hitchner et al., 2020; Kuroda, Caputo & DeGrado, 2009; Matsuzaki, 2009; Saint Jean et al., 2018). However, the relative lack of study on the L-subfamily of ponericins leaves many unanswered question structure-activity and sequence-activity questions. Using a combination of spectroscopic and microbiological approaches, five variants of L1 were characterized for in-solution aggregation, lipid binding, secondary structure formation, antimicrobial activity, and membrane permeabilization.

## Materials and Methods

### Materials:

Lipids 1,2-dioleoyl-sn-glycero-3-phosphocholine (DOPC; PC) 1,2-dioleoyl-sn-glycero-3-phospho-(10-rac-glycerol) (DOPG; PG), and 1-palmitoyl-2-oleoyl-sn-glycero-3-phosphoethanolamine (POPE; PE) were purchased from Avanti Polar Lipids (Alabaster, Alabama), and stored as stocks in chloroform at −20 C. Lipids were prepared as 100% DOPC, 3:1 DOPC:Cholesterol (PC:Chol), 3:1 DOPC:DOPG (PC:PG), or 3:1 POPE:DOPG (PE:PG). Isopropyl β-D-1-thiogalactopyranoside (IPTG) (Chem-Impex Int’l INC.), orthonitrophenyl-β-galactoside (ONPG) (Research Products International Co.), nitrocefin (Biovision, Milpitas, California). All other chemicals and reagents were from Thermo Fisher Scientific (Waltham,Massachusetts), VWR (Radnor, Pennsylvania), or Sigma-Aldrich (St. Louis, Missouri).

Peptide variants L1A and L1F were synthesized using standard FMOC-chemistry in-house. The parent peptide L1 along with L1-V, L1-I, and L1-L were purchased from Synthetic Proteomics (Carlsbad, California). Using a Zorbax C3 column, all peptides were purified via RP-HPLC eluted by a linear gradient of water to acetonitrile (both supplemented with 0.1% TFA). Peptide identity was confirmed using MALDI-TOF mass spectrommetry. HPLC fractions containing the peptide were collected, pooled, lyophilized, and stored a at −20 C. Peptides were dissolved in H_2_O:ethanol 3:1 prior to use. Each peptide stock was made to a final concentration of 150 −200 μM then stored at 4 C.

To form small unilamellar vesicles (SUVs) sonication or ethanol dilution methods were implemented (Caputo & London, 2019). In all cases, lipid vesicle preparation was the same. Depending on the lipid composition, appropriate volumes of lipids in chloroform were mixed in a glass tube and then allowed to dry under N_2_ gas for 15 minutes. The lipid films were then placed in a vacuum desiccator and allowed to dry further under a vacuum for 1 hour. For vesicles formed through ethanol dilution methods, 10 μL of pure ethanol was added to the film, and then vortex until the dissipation of the film. While continuing to vortex, the appropriate amount of PBS was added. For vesicles formed using the method of sonication, PBS was added directly to the tube while being vortexed to create multilamellar vesicles (MLVs). The MLV suspension was covered with parafilm and then placed in a high-powered bath sonicator (Avanti Lipids) and subjected to sonication for 20 minutes to create SUVs.

All images were created using Microsoft Excel and the Daniel’s XL toolbox plugin for high resolution images (Kraus, 2014).

### Fluorescence spectroscopy:

All fluorescence spectroscopy experiments were preformed using a JY-Horiba Fluoromax4 instrument. Excitation and emission slit widths were kept consistent at 2.5 nm. Semi-micro quartz cuvettes were utilized for each experiment. The following formula was used to calculate a spectral barycenter for each experiment:

(1) $$B=\frac{\sum \lambda *i}{\sum i}$$

where B is the barycenter, *λ* is the wavelength, and *i* is the intensity at the specified wavelength. An entire emission spectrum from 300-400 was used the carry out the calculation of barycenter.

Lipid binding protocols from previous experiments were used as a basis for the following reported data. Peptide samples were prepared at final concentration of 2μM in PBS buffer ( 150 mM NaCl, 50 mM Na_2_HPO_4_, pH 7.0) and titrated with lipid vesicle stock (usually [1.0] mM total lipid). The standard for all binding assays is λ_ex_ = 280 nm and λ_em_ = 300 to 400 nm. A sample containing only PBS and no peptide was used as a background to correct spectra after each addition of vesicles.

Red edge excitation shift (REES) experiments utilized excitation at 6 different wavelengths (λ_ex_ = 280, 290, 295, 300, 305, and 307 nm) and the same emission range of 310 to 410 nm. Samples were similarly prepared as above. A blank only containing PBS buffer was used as background to correct the spectra after each addition.

Trichloroethanol (TCE) quenching was monitored by titration of samples with 10 μL aliquots of 10M TCE (Alfa Aesar, Haverhill, Massachusetts) into peptide samples prepared as described above. After each addition of TCE, Trp fluorescence intensity was measured using λ_ex_ = 280 nm and λ_em_ = 340. Corrections to the emission intensity were made for background subtraction and dilutions. The slope of the linear best fit equation to the data (eqn 2) was used as the Stern-Volmer quenching constant (Ksv)

(2) $$\frac{F0}{F}=1+Ksv[Q]$$

where F_0_ is the fluorescence intensity in the absence of quencher, and F is the fluorescence intensity in the presence of a given concentration of quencher [Q].

Acrylamide quenching was monitored by titration of samples with 10 μL aliquots of 4M acrylamide titrated into peptide samples prepared as described above. These samples contained either [0] or [0.25] mM total lipid. After each addition of acrylamide, Trp fluorescence intensity was measured using λ_ex_ = 295 nm and λ_em_ = 340. Corrections to the emission intensity were made for background subtraction, dilutions, and inner filter effects in the excitation path as described previously. The same method to calculate Ksv was used as above.

### Circular Dichroism Spectroscopy:

Circular Dichroism (CD) spectra were collected via Jasco J-810 spectropolarimeter. CD experiments with samples containing SUVs with 200 μM total lipid and 3 μM peptide in 0.1 x PBS buffer. Each measurement was an average of at least 64 scans and was corrected by subtracting background spectra that did not contain peptide.

### Minimal Inhibitory concentration/minimal bactericidal concentration:

Six different strains of bacteria were streaked on LB-Miller agar plates from a frozen glycerol stock derived from the original samples shipped from CGSC (Coli Genetic Stock Center, Yale University) or ATCC (American Type Culture Collection) (*Escherichia coli* D31 CGSC 5165 (Burman, Nordstrom & Boman, 1968), *Staphylococcus aureus* ATCC 35556. *Pseudomonas aeruginosa* PA-01 ATCC 47085, *Acinetobacter baumannii* ATCC 19606). For each of the strains a liquid overnight culture was prepared using a single colony into fresh LB broth and placed in a shaking incubator for ~ 18 hours at 37 C at 225 rpm. After the incubation period, a 1:200 dilution was made in fresh LB broth and then used for experimentation. Minimal Inhibitory Concentration (MIC) determinations were performed using mid-log phase bacteria diluted to 5 x 10^5^ CFU/mL. Next, 90 μL of this diluted culture was then pipetted into a sterile 96-well plate that contains serially diluted aliquots of the peptide variants to consist of a total volume of 100 μL. This plate was then incubated for 18 hours at 37 C. Bacterial growth was determined by taking an OD_600_ measurement using a Spectramax M5 multimode plate reader post incubation. Minimal Bactericidal Concentration (MBC) was performed by taking 1 μL of the culture from the MIC plate and then pipetting it on to a fresh LB agar plate. After plating this culture, the plate was then incubated overnight at 37 C. MBC was determined by the growth or the lack of growth from each well on the agar plate.

### Inner membrane permeabilization:

A single colony of *E.coli D31* was inoculated into LB broth and placed in a shaking incubator and set to 225 rpm at 37 C for ~ 18 hours. A 1:250 dilution was made using the overnight culture into fresh LB broth. To induce the expression the expression of β-galactosidase, a final concentration of 100mM of IPTG was introduced. This dilution plus the IPTG was placed back into the shaking incubator for about 2 hours until an OD_600_ of 0.2-0.4 was achieved.

A 96 well plate was prepared with the following solutions in order: 10 μL of each peptide variant 2-fold serially diluted down the plate with the exception of the final row (used as a negative control 10 μL of 0.01% acetic acid), 56 μL Z-buffer (60mM Na_2_HPO_4_, 40mM NaH_2_PO_4_, 10mM KCl, 1mM MgSO_4_, 50mM β-mercaptoethanol pH 7), 19 μL of the *E. coli* culture, and 15 μL of 4 mg/ml ONPG that was prepared in Z-buffer added immediately before the assay began. Absorbance measurements wer taken at 420 nm every 5 minutes for a total run time of 90 minutes. The detergent cetyltrimethylammonium bromide (CTAB) was used as a positive control. The data presented is an average of three replicates.

### Outer membrane permeabilization:

A single colony of *E.coli D31* was taken from an LB agar plate and inoculated into fresh LB broth with 100 μg/mL ampicillin (LB-Amp), then place in a shaking incubator at 37 C (225 rpm) for ~ 18 hours. After the incubation period 100 μL was taken from the culture and diluted with 25 mL of fresh LB-Amp. The dilution was further incubated in the same conditions until an OD_600_ of 0.2 to 0.4 was obtained. Once the specified OD_600_ was reached the culture was then centrifuged at 2500 rpm for 15 minutes in a tabletop clinical centrifuge. The supernatant was discarded and then an equal amount of PBS (100 mM NaH_2_PO_4_, 200 mM NaCl, pH7) was used to resuspend the pellet.

Experimental samples were prepared in a 96-well plate in the order as follows: 10 μL of the six peptide variants that were serially diluted starting a concentration of 15 μM with the exception of the last row (used as a negative control 10 μL of 0.01% acetic acid). 80 μL of the resuspended bacteria culture, and 10 μL of 50 μg/mL nitrocefin in PBS. The absorbance was recorded at 486 nm in 5-minute intervals for a total run time of 90 minutes immediately following the addition of nitrocefin. The antimicrobial agent polymyxin B was used as a positive control. The values presented are the average of three replicates.

### Hemolysis:

Hemolysis on Sheep red blood cells (RBCs) was used to quantify the destabilization of membranes by leakage of hemoglobin. Sheep blood was obtained through Hemostat Laboratories and stored at 2 – 8 C. A 7 mL aliquot of whole sheep blood was mixed with 7 mL of sterile PBS at pH 7.4. The solution was sedimented via centrifugation for 7 minutes at 2500 rpm. Supernatant was removed and the pellet was resuspended to the original volume of 14 mL. This was repeated three times and the final pellet was resuspended to 14 with PBS. Next, 90 μl of the cell suspension was pipetted into all wells of a conical bottom 96-well plate. 10 μL of serially diluted peptide variant or the detergent TX-100 (positive control) were added to the wells prior to addition of the RBC’s. The plate was covered and left in a shaking incubator at 37 C at 150 rpm for 1 hour. Post incubation the plate was then centrifuged for 10 minutes at 200 rpm at 4 C. 6 μL of the supernatant was added to 94 μl of fresh PBS in a flat bottom plate. The absorbance of the plate was taken at 415 nm using a Spectramax M5 multimode plate reader. By using the absorbance of each well compared to the wells without additive and those with Triton-X 100 a percentage of hemolysis was calculated. The data presented are the average of three replicates.

## Results

### Peptides

**Fig. 1:**
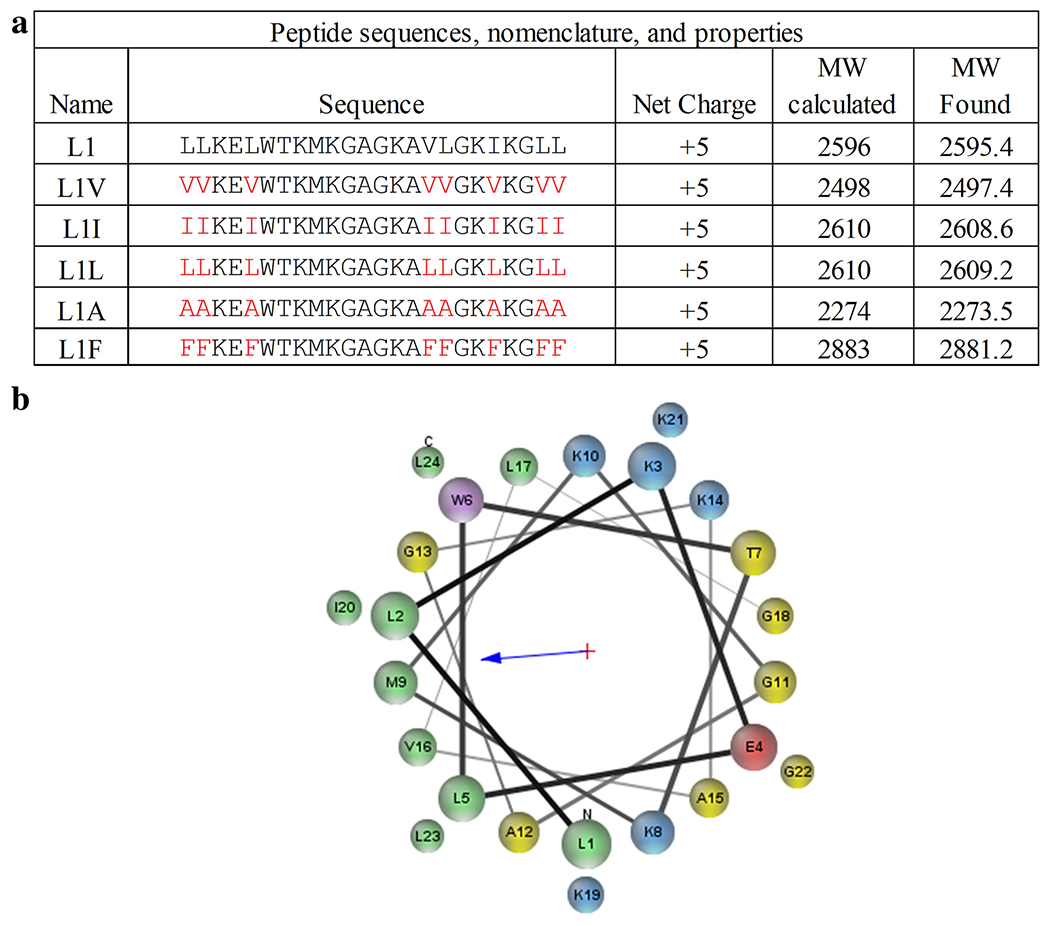
Peptide sequences and helical wheel diagram – (A) Peptide sequences and relevant physico-chemical properties. (B) Helical wheel diagram of L1. Cationic residues are shown in blue, anionic in red, hydrophobic in green, polar uncharged in yellow, and aromatic are in purple. Helical wheel representation was made using MPEx (Snider et al., 2009)

All peptides in this study were synthesized via solid-*phase* peptide synthesis approaches and purified by reversed-phase HPLC. The sequences used in this study and relevant physico-chemical properties are shown in Figure 1A. As the L1 peptide has previously been shown to adopt an α-helical conformation when bound to lipid bilayers, a helical wheel diagram of the parent L1 sequence is shown in Figure 1B.

### Antimicrobial activity

The ability of L1 and the hydrophobic variants to act as antimicrobials was investigated using the Minimal Inhibitory Concentration (MIC) and Minimal Bactericidal Concentration (MBC) assays as previously described (Wiegand, Hilpert & Hancock, 2008). The MIC is determined as the lowest concentration of peptide required to inhibit bacterial growth in solution, while the MBC is the lowest concentration required to act in a bactericidal mechanism (as opposed to bacteriostatic) and is determined by plating MIC cultures on antimicrobial-free media. Peptides were screened against *E. coli, S. aureus, A. baumannii, P. aeruginosa, B. subtilis*, and *K. pneumoniae.* The results are shown in Table 1. The highest concentration tested was 15 μM peptide, thus any samples that did not exhibit bacterial growth inhibition at this concentration the MIC is listed as > 15.

### Solution aggregation properties

Considering that the first step in the mechanism of action of many AMPs is the binding to the bacterial cell surface, any aggregation or oligomerization of peptides can impact the binding equilibrium. If amino acid substitutions promote the aggregated state, this will then reduce the amount of free peptide available to bind the bacteria and exert antimicrobial activity. This is especially relevant in reference to hydrophobic substitutions as hydrophobicity of a peptide is a significant driver of peptide aggregation in solution (Rani, Kuroda & Vemparala, 2020; Vaezi et al., 2020; Zapadka et al., 2017) .

**Fig. 2:**
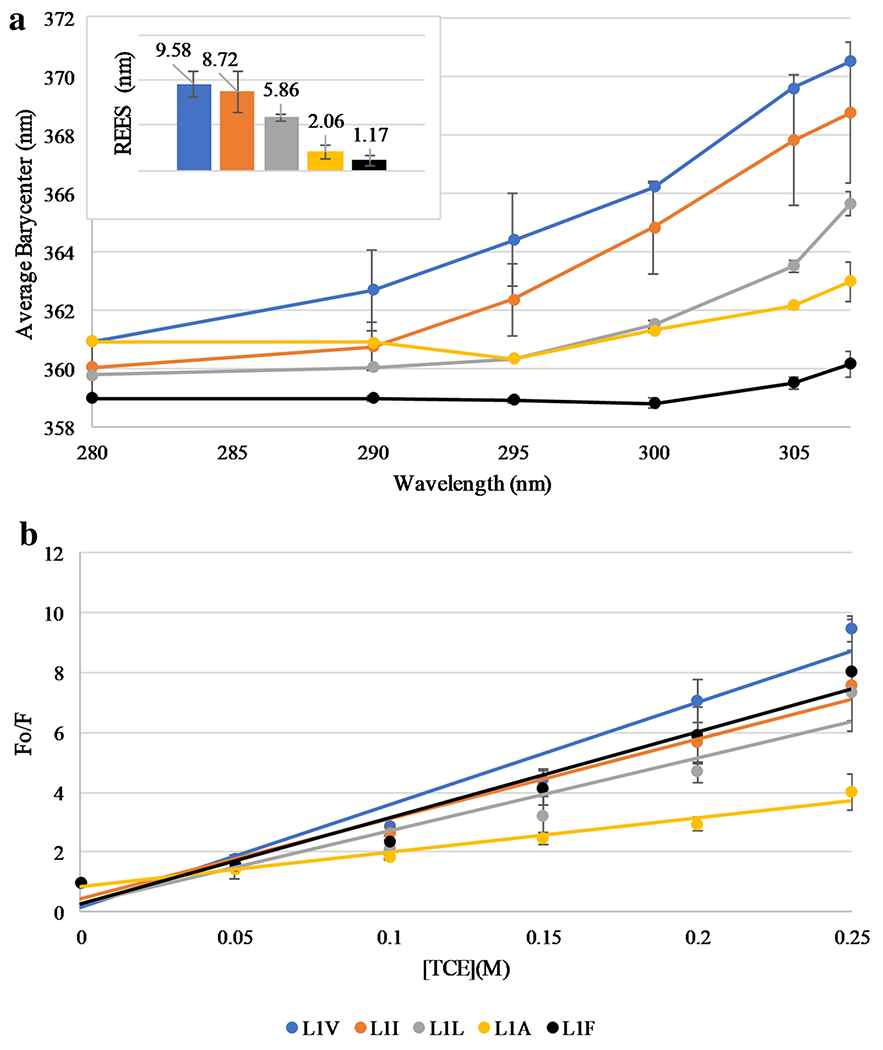
Peptide solution aggregation assays—(A) Red Edge Excitation shift (REES) and (B) Trichloroethanol (TCE) quenching. The inset in panel A represents the total shift (nm) for each peptide. In both panels L1V is shown in light blue, L1I orange, L1L grey, L1A yellow, and L1F black. Peptide concentration was 2 μM in all samples. All data are averages of three replicates and SD is represented by error bars. In some cases the error bars are smaller than the size of the symbol

Aggregation of peptides was assessed utilizing the intrinsic fluorescence properties of the Trp residue in the L1 sequence. Since L1 contains a single Trp, this residue can serve as a reporter for a variety of peptide characteristics, most notably the environment that the Trp, and thus the peptide, is sampling. Differences in the side chain motion/dynamics of the Trp residue and the local environment around the Trp can be observed by monitoring the red-edge excitation shifts (REES). This approach monitors shifts in the emission spectrum as a function of varying the excitation wavelength which is directly influenced by the mobility and solvent accessibility of the Trp side chain (Haldar, Raghuraman & Chattopadhyay, 2008; Raghuraman & Chattopadhyay, 2006). When a Trp is exposed to the aqueous milieu and is free to sample rotational conformations, no REES is exhibited, while if the Trp is constrained or shielded from bulk water REES is observed as the excitation wavelength is increased. The REES results are shown in Figure 2A. Notably, all peptides exhibited some degree of REES, with L1V exhibiting the strongest REES of ~10 nm. Conversely, the L1F peptide exhibited the least REES, only ~2 nm. The total shift for each peptide is shown in the inset of Figure 2A.

The environment of the L1 Trp residue was further probed using fluorescence quenching by trichloroethanol (TCE) which selectively exerts quenching on Trp residues which are shielded from the aqueous milieu, such as at the interior of a peptide aggregate. The results are shown in Figure 2B, with the Ksv values for the quenching shown in Supplemental Table 1. The majority of the peptides showed similar extent of quenching by TCE except the L1A variant. The Trp quenching in this peptide sample was markedly lower than the others. However, considering the reduced overall hydrophobicity of this peptide and thus higher aqueous solubility, decreased TCE quenching is not surprising. Importantly, the L1V showed the most quenching, consistent with the REES results.

### Peptide-lipid interactions

**Fig. 3:**
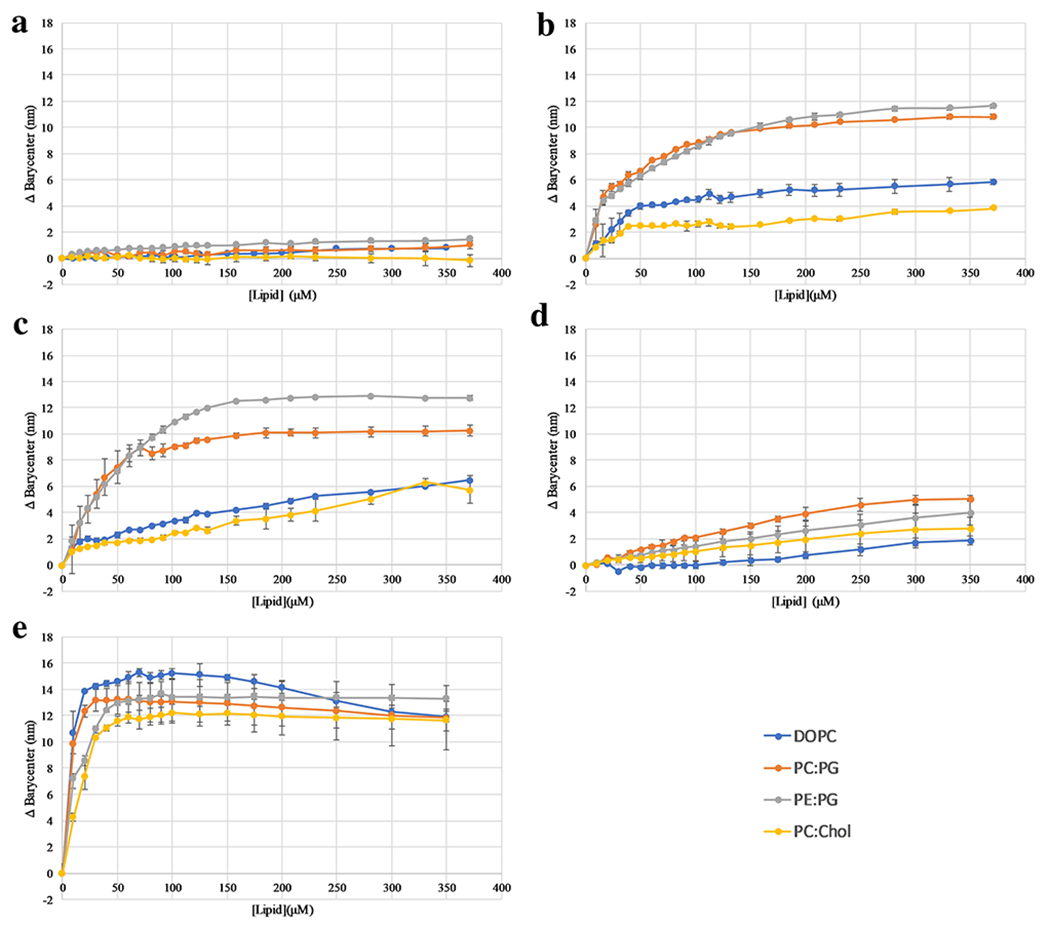
Peptide binding to lipid vesicles—Peptide binding was assayed using Trp fluorescence by titrating SUVs into a sample containing 2 μM peptide in PBS buffer. After each addition of vesicles the spectrum was recorded and the spectral barycenter and Δ Barycenter were calculated. Peptide variants were (A) L1V, (B) L1I, (C) L1L, (D) L1A, (E) L1F. In all panels blue represents PC, orange 3:1 PC:PG, grey 3:1 PE:PG, and yellow 3:1 PC:Chol. All data are averages of 3 replicates and SD is represented by error bars. In some cases the error bars are smaller than the size of the symbol

Considering that the first step in the mechanism of action of many AMPs is the binding to the bacterial cell surface, binding to model lipid vesicles was performed. These experiments exploited the inherent environmental sensitivity of Trp as a reporter for binding to bilayers. In aqueous solution, the Trp fluorescence exhibits a red shifted emission barycenter, while when bound to bilayers and thus in a more non-polar environment the Trp emission blue-shifts to lower wavelengths (Ladokhin, Selsted & White, 1997; Savinov & Heuck, 2017). The impact of lipid composition on binding to model vesicles was investigated using a simple vesicle titration experiment in which Trp fluorescence was measured after addition of lipid vesicles. The change in the emission barycenter compared to that in absence of vesicles was plotted as a function of total lipid concentration Figure 3 and selected spectra are shown in Supplemental Figure 1 (S1). Lipid composition of vesicles was either 100%DOPC, 3:1 DOPC:DOPG, 3:1 POPE:DOPG, or 7:3 DOPC:Cholesterol. The results show varied behavior that is both peptide and lipid composition dependent. The L1L and L1I variants show significantly larger shifts in Trp emission barycenter when binding PG-containing bilayers, indicating that electrostatic forces are a strong driver in the bilayer association of these peptides. In contrast, L1F, L1V, and L1A showed no significant preference for anionic vs. zwitterionic bilayers. However, only the L1F showed significant shifts in barycenter with the addition of lipid while L1A and L1V did not. The L1F results indicate that binding in this case is driven more strongly by hydrophobic interactions independent of the electrostatic contribution. The L1A and L1V results indicate little interaction with the bilayer, or perhaps only very shallow interactions with the surface of the bilayer which does not result in a significant environmental change around the Trp.

**Fig. 4:**
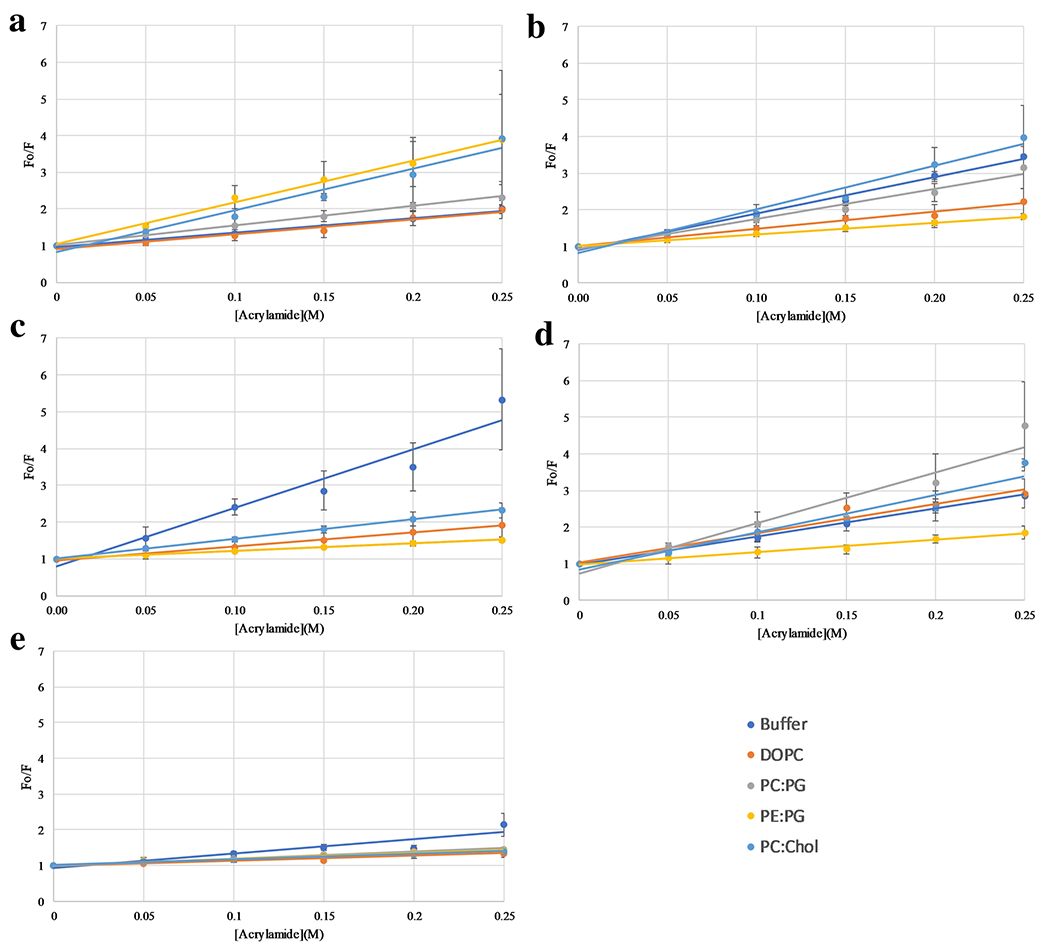
Acrylamide quenching of peptides in the absence and presence of lipid vesicles. Samples contained either no lipid vesicles (dark blue), DOPC vesicles (orange ), PC:PG vesicles (grey), PE:PG vesicles (yellow), or PC:Chol vesicles (light blue). All samples contained a peptide concentration of 2 μM and lipid concentration of 250 μM for those that contained lipid vesicles. (A) L1V, (B) L1I, (C) L1L, (D) L1A, (E) LIF.Data is corrected for inner filter effects, dilution, and are background subtracted. Data represents an average of three replicates with SD shown by the error bars. In some cases the error bars are smaller than the size of the symbol

In order to gain more insight into the bilayer interactions, fluorescence quenching approaches were utilized. The peptides were exposed to the aqueous quencher acrylamide in solution or when bound to vesicles. Acrylamide is known to strongly quench Trp fluorescence in aqueous environments but does not exert the quenching effect if the Trp is buried in the nonpolar core of the bilayer (Caputo & London, 2019). Thus, peptide Trp exposure to the aqueous milieu can be gauged by the extent of quenching by acrylamide. Stern-Volmer quenching plots were created to analyze the data and Ksv (slopes of a linear fit to the data) are shown in Figure 4 and Table 2. The trends in this data are consistent with the barycenter shift data in Figure 3. In the case of L1L and L1I, there was a significant reduction in quenching between the solution (free) state of the peptide and when bound to anionic vesicles, but a much smaller reduction if any when interacting with zwitterionic vesicles. This is consistent with a more deeply buried Trp in anionic bilayers. The L1V and L1A exhibited differential behavior in this experiment with L1V showing a reduction in quenching with vesicles, indicating some interaction while the L1A did not exhibit significant reductions indicating that the Trp is significantly exposed under all conditions. Importantly, the L1F was the only peptide to exhibit extensive reductions in quenching when bound to PC:Chol vesicles, consistent with the strong binding to both anionic and zwitterionic vesicles.

### Peptide secondary structure

**Fig 5:**
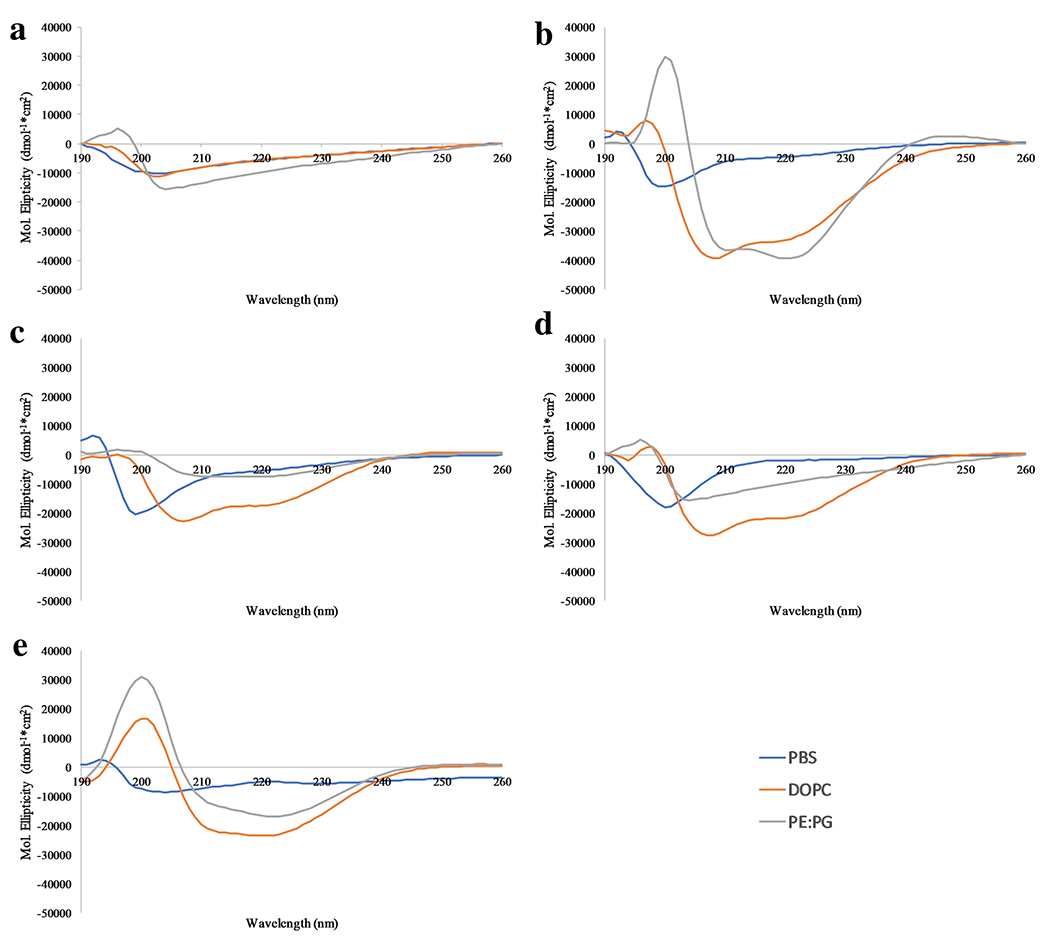
Circular Dichroism spectroscopy – Spectra of all peptide variants (A) L1V , (B) L1I, (C) L1L, (D) L1A, and (E) L1F. Samples contained a final peptide concentration of 3μM and those with lipid vesicles contained 200 μM total lipid. Spectra of all peptides were collected in 10X diluted PBS (blue), DOPC vesicles (orange), and 3:1 PE:PG vesicles (grey). All data represent the average of 64 scans with correction of background spectra lacking peptide.

While many venom peptides that act as AMPs adopt helical secondary structures, it is also known that amino acids have differing propensities to form alpha-helical structures. Circular dichroism (CD) spectroscopy was used to characterize the secondary structure of the peptides in the absence and presence of lipid vesicles. The CD spectra were recorded of the peptides in buffer or in the presence of DOPC vesicles. DOPC was selected as all peptides interact with these bilayers to some extent and the favorable spectroscopic profile of DOPC vesicles compared to those containing PG. The CD results are shown in Figure 5. In buffer alone, all peptides exhibited canonical spectral signatures consistent with disordered secondary structure except L1V. When in the presence of lipid vesicles, L1F, L1I, and L1A clearly adopt α-helical conformations in both types of lipid vesicles while L1L appears to be helical in DOPC vesicles but exhibits less helical content when bound to PE:PG vesicles. Interestingly, there was almost no difference in CD spectra for the L1V peptide in the presence or absence of vesicles. The L1V peptide exhibited a strong negative band at ~204 nm which is inconsistent with disordered structures, α-helix, or β-strand (Greenfield, 2006).

### Membrane permeabilization

**Fig. 6:**
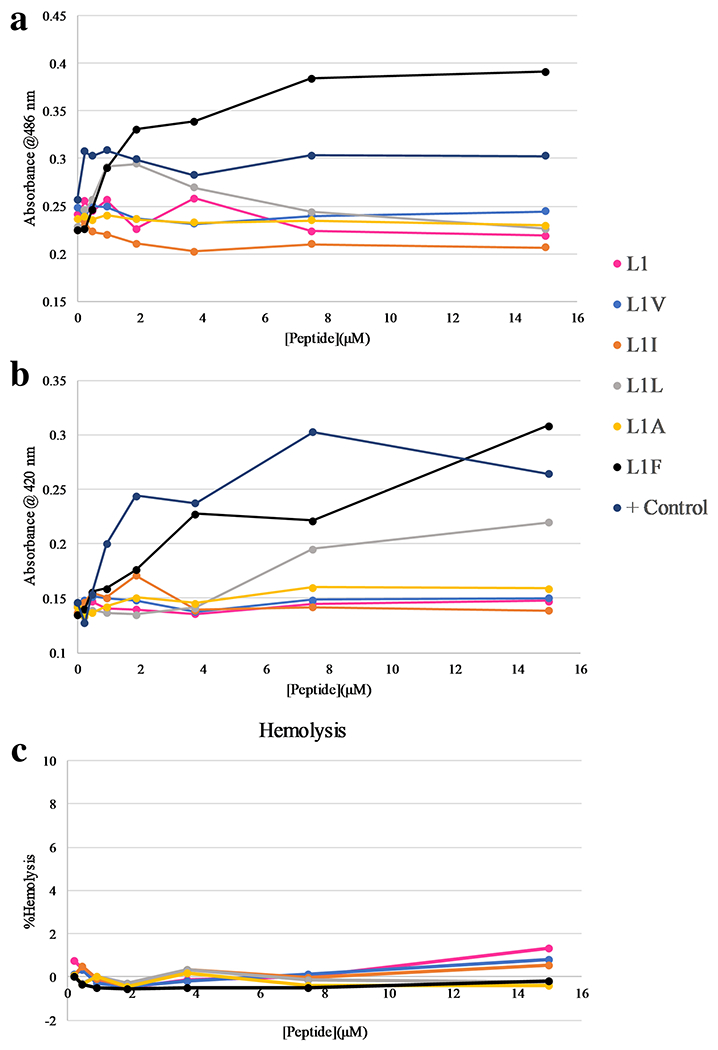
Membrane permeabilization assays. Permeabilization of of in-tact (A) *E. coli* outer membrane, (B) *E. coli inner* and (C) ovine RBCs (hemolysis). For all panels: L1 (pink), L1V (blue), L1I (orange), L1L (grey), L1A (yellow), and L1F (black). In (A) and (B) the positive controls are shown in dark blue, representing polymyxin B in (A) and CTAB in (B). The data shown in both panels (A) and (B) are after 30 minutes of exposure to the peptide/control. Data sets in C, were normalized to values based on untreated RBCs (0% hemolysis) and RBCs treated with Triton X-100 (100% hemolysis). All data are averages of three replicates and SD represented by error bars. In all cases, the error bars are smaller than the size of the symbols

As the mechanism of action of the L1 venom peptide is likely a membrane disruptive mechanism, the effect of the hydrophobic substitutions on membrane permeabilization was investigated using *E.coli* and ovine red blood cells (RBCs). The permeabilization of the *E. coli* outer membrane was assayed using an enzyme-substrate pair in which the enzyme (β-lactamase) is located in the periplasmic space while the substrate (the chromogenic nitrocefin) is added to the exterior of cells. Normally, nitrocefin has limited permeability across the bilayer, but as peptides or other membrane active agents disrupt bilayer integrity, the nitrocefin can more easily cross the outer membrane into the periplasm where it is hydrolyzed by β-lactamase into a colored product. A snapshot of the results after 30 minutes of exposure are shown in Figure 6A, with full time courses shown in Supplemental Figure 2 (S2). In this assay, L1L and L1F showed increased permeability of the outer membrane, but L1F induced permeabilization more quickly and at lower concentrations that L1L. Notably, several other peptides induced some extent of delayed permeabilization at the highest concentrations tested (Supplemental Figure S2), however L1I, despite the similar biophysical behavior demonstrated earlier, exhibited no membrane disruption above background. Similarly, the permeabilization of the *E. coli* inner membrane was assessed using a cytoplasmic enzyme, β-galactosidase, and a chromogenic substrate, ONPG. A snapshot of the results after 30 minutes of exposure are shown in Figure 6B, with full time courses shown in Supplemental Figure 3 (S3). In this case, only L1L and L1F exhibited any inner membrane permeabilizing activity, regardless of the time of peptide exposure.

Hemolytic activity of venom peptides and, more broadly AMPS in general, has often been a first approximation toward assessing potential for further development. Highly hemolytic peptides are unlikely to become useful clinical therapeutics due to the toxicity to the host. Venom peptides in particular often suffer from high hemolytic activity due to the evolutionary nature of these molecules and the original purpose in the source organism. Additionally, it has been demonstrated that the overall hydrophobicity of antimicrobial polymers directly impacts the cytotoxic and hemolytic activity of these molecules, thus screening these hydrophobic-substituted peptides for hemolysis was an important step. The peptides we co-incubated with freshly isolated ovine red blood cells and peptide-induced hemolysis was determined by comparing hemoglobin leakage from treated cells to that of untreated cells as well as cells treated with the detergent Triton X-100 as a positive control. The results of hemolysis assays are shown in Figure 6C. The results indicate that none of the L1 variants induced any measurable hemolysis up to 15μM peptide. These results are consistent with the previous results from on the L1 parent sequence and other sequence variants of L1 (Senetra, Necelis & Caputo, 2020a).

## Discussion

The work presented herein describes an investigation into hydrophobic amino acids and how a uniform substitution strategy impacts the biophysical and microbiological activity of the venom derived peptide Ponericin L1. Overall the behavior of the peptide can be dramatically impacted by the substitution of hydrophobic residues in different ways, depending on the identity of the substitutions.

While many AMPs have evolved to selectively target bacterial cell membranes over host cell membranes, the same selective pressure is not present for venom peptides. Notably, venom peptides generally act as an offensive weapon against predators or prey, and would generally benefit from being more broad-spectrum in activity in this role (Aili et al., 2014; Johnson et al., 2010; Orivel et al., 2001). However, any hope of development of a membrane-active peptide into a clinical therapeutic must have the ability to discriminate between host cell membranes and bacterial cell surfaces. The biophysical results clearly show differences in affinity to model lipid vesicles. The L1L, L1I, and L1F all show a significant change in emission barycenter upon exposure to anionic lipid vesicles. This indicates that the peptides are interacting with the vesicles and the Trp residue undergoes a change in local environment into a more non-polar environment. This is consistent with the physical properties of the peptides as these three molecules have a net positive charge and are the three most hydrophobic peptides when evaluated using the Wimley-White hydrophobicity scale (Wimley & White, 1996). However, the L1F is the only peptide to bind zwitterionic lipid vesicles over similar concentration ranges and resulting in similar extent of spectral shift (~14 nm). This is likely driven by the increased hydrophobicity of the molecule compared to L1L and L1I. On the other hand, the L1A and L1V peptides did not show nearly as much binding to the vesicles. While these are less hydrophobic in general, the L1V exhibited the least shift despite being more hydrophobic than L1A.

The interaction with anionic model lipid bilayers appears to follow the same pattern as the MIC results. The weakest binding L1A and L1V peptides showed no antimicrobial activity at the highest concentrations tested (15μM). Conversely, L1L, L1I, and L1F exhibited broad spectrum activity against both Gram-positive and Gram-negative strains. Similarly, these three peptides exhibited the least quenching by acrylamide. Taken together, the binding to bilayer surfaces and the ability to interact with the nonpolar core of the bilayer appears to be a good screening tool for these peptides. Beyond this, the hydrophobic component in binding appears to have a threshold effect on both the blue-shift of the Trp fluorescence and the antimicrobial activity. Spectroscopically, the more hydrophobic peptides likely partition deeper into the bilayer, burying the Trp in a more nonpolar environment shield from acrylamide. Similarly, this partitioning depth has been shown by our group and others to be a contributor to antimicrobial activity (Hitchner et al., 2021; Palermo & Kuroda, 2009; Palermo & Kuroda, 2010; Saint Jean et al., 2018). However, the correlation of binding to antimicrobial activity is not universal. Prior work from our group on a different peptide, C18G-His, demonstrated that peptide exhibited a strong blue shift upon binding at neutral pH but did not have strong antimicrobial activity at that pH regime (Hitchner et al., 2019). Thus, while this correlation between binding and activity is suggestive for L1, care must be taken to ensure that multiple avenues are examined when using experiments to guide design of novel peptide antimicrobials.

An interesting result from this work is the complete lack of activity exhibited by L1V. This was somewhat surprising due to the hydrophobic nature of the peptide coupled with the strong cationic net charge. An examination of the data indicates that this is likely driven by structure formation in the peptide while in solution. The L1V peptide exhibited the highest quenching by TCE in solution as well as exhibiting the highest degree of red-edge shift, indicative of solution interactions in the peptide. The CD spectra show no difference in the absence or presence of lipid vesicles, supporting the binding results that L1V does not interact with bilayers. Additionally, the CD spectra for L1V exhibit a minimum ~205nm, somewhat red-shifted for a disordered structure and blue-shifted for a β-strand. However, similar spectra have been reported for β-turn AMPs by several different groups (Dong et al., 2012; Gottler et al., 2008). This formation of some β-strand structure is consistent with valine having a lower helical propensity than the other residues examined (Neil & DeGrado, 1990; Nick Pace & Martin Scholtz, 1998). The structure prediction algorithm, iTasser, was used to generate predicted structures of L1L and L1V which also support the formation of β-strand formation in L1V (Supplemental Figure 4) (Yang & Zhang, 2015). These predictions only model the proposed structure in solution, but appear to also correlate well to the bilayer-bound conformations observed in DOPC vesicles. Taken together, the data support the hypothesis that the activity of the peptides is a balance between hydrophobicity, cationic charge, and secondary structure.

Finally, the lack of hemolytic activity of these peptides is striking. While not totally unexpected based on prior results with L1-based sequences with substitutions to the cationic residues, it is exciting that these molecules retain this beneficial property even upon hydrophobic substitutions. Prior studies with AMP-mimetic polymers show a clear relationship between overall hydrophobicity and cytotoxicity and/or hemolytic activity (Mortazavian et al., 2018; Sovadinova et al., 2011). In those works, the Kuroda group performed hemolysis assays on a series of heteropolymers in which the fraction and type of hydrophobic monomers were varied. These results clearly showed as the fraction of hydrophobic monomers increased, or the hydrophobicity of the monomers increased (methyl vs. butyl), the hemolytic activity also increased (Mortazavian et al., 2018; Sovadinova et al., 2011). However, it appears in the L1 background that even the most hydrophobic substitutions (L1L and L1F) do not cross the threshold for inducing hemolytic activity. Speculatively, the strong interactions between L1F and zwitterionic bilayers may indicate that the hemolytic threshold is not far off, as it indicates the peptide should be able to bind to RBC or other zwitterionic mammalian membranes.

## Conclusions

Taken together, the results demonstrate the Ponericin L1 peptide is a flexible peptide platform for antimicrobial development. Importantly the results demonstrate the importance of the secondary structure of the peptide to maintain antimicrobial activity and beneficial binding properties. The peptides appear to act through a membrane disruptive mechanism, but it is unclear if there are additional mechanisms of action post-permeabilization. Importantly, there may be additional insights to be gained regarding mechanism of action and efficacy from the context of the natural venom cocktail. Considering the natural cocktail includes multiple sequence variants, including several that are enriched in aromatic amino acids, there may be an evolved synergy between the peptides to exert toxicity. An in depth combinatorial study of these sequences may discover combinations that enhance efficacy and maintain the promisingly low hemolytic profile exhibited in Ponericin L1.

## Supplementary Material

1759789_Sup_info.

## Figures and Tables

**Table 1:** Minimal Inhibitory Concentration / Minimal Bactericidal Concentration (μM)

	*S. aureus*		*E. coli*		*A. baumannii*		*P. aeruginosa*		*B. subtilis*		*K. pneumoniae*	

	MIC	MBC	MIC	MBC	MIC	MBC	MIC	MBC	MIC	MBC	MIC	MBC
L1	7.50	15	0.94	0.94	1.875	3.75	>15	>15	0.47	0.94	3.75	>15
L1V	>15	>15	>15	>15	>15	>15	>15	>15	>15	>15	>15	>15
L1I	15	15	0.47	3.75	1.88	1.88	>15	>15	0.47	0.47	7.5	7.5
L1L	1.88	3.75	0.47	0.94	0.94	0.94	15	>15	0.47	0.94	3.75	>15
L1A	>15	>15	>15	>15	>15	>15	>15	>15	>15	>15	>15	>15
L1F	0.94	1.88	0.94	>15	0.47	0.938	7.5	>15	1.88	1.88	1.88	7.5

**Table 2:** Acrylamide quenching (Ksv [M^−1^])

	PBS	PC	PCPG	PEPG	PC/Chol
**L1V**	12.14 ± 1.59	4.18 ± 0.15	16.86 ± 6.47	5.64 ± 0.38	12.19 ± 1.54
**L1I**	10.5 ± 0.67	4.9 ± 1.51	8.72 ± 2.09	3.39 ± 2.25	12.65 ± 0.63
**L1L**	4.26 ± 0.97	4.08 ± 2.10	3.51 ± 0.17	2.22 ± 4.04	8.92 ± 0.94
**L1A**	8.19 ± 0.90	8.51 ± 0.46	10.9 ± 0.26	3.59 ± 0.58	10.79 ± 0.27
**L1F**	4.18 ± 4.79	1.57 ± 3.49	2.06 ± 0.97	1.80 ± 0.77	1.63 ± 0.20
