# Supplementary material for: Investigation of the Role of Hydrophobic Amino Acids on the Structure-Activity Relationship in the Antimicrobial Venom Peptide Ponericin L1
Source: J Membr Biol. Author manuscript; Available in PMC 2023 Oct 1. (PMC9114170; doi:10.1007/s00232-021-00204-y)
Supplement: 1759789_Sup_info. [file NIHMS1759789-supplement-1759789_Sup_info_.pdf]

Supplemental Information: Investigation of the Role of Hydrophobic Amino Acids on the Structure-Activity Relationship in the Antimicrobial Venom Peptide Ponericin L1

Nicholas P. Schifano[1] and Gregory A. Caputo[1,2,*]

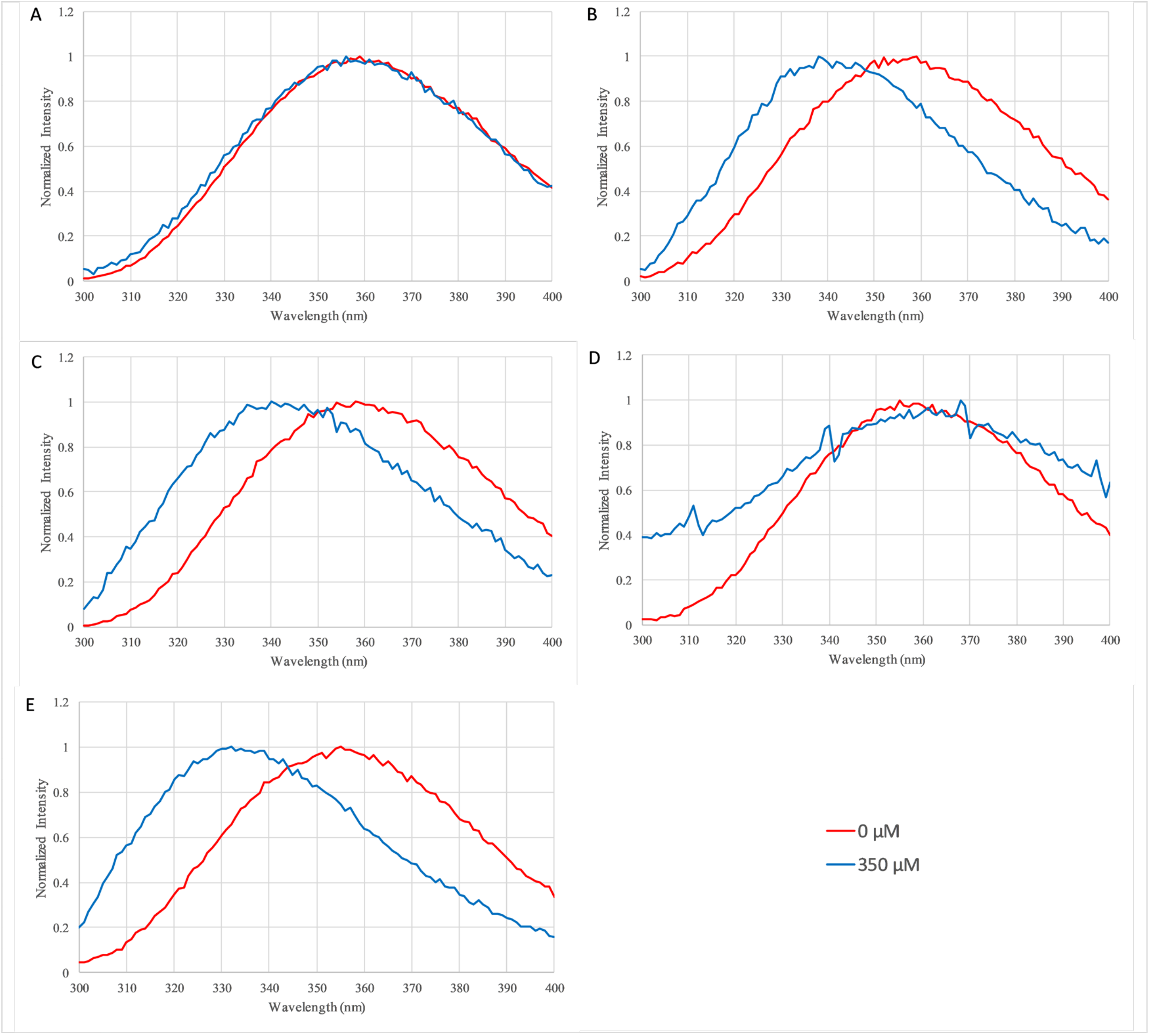

Supplemental Figure S1 – Normalized emission spectra of peptides in the absence (red) or presence (blue) of PC:PG SUVs. (A) L1V, (B) L1I, (C) L1L, (D) L1A, (E) L1F. In all panels, peptide concentration was 2 µM.

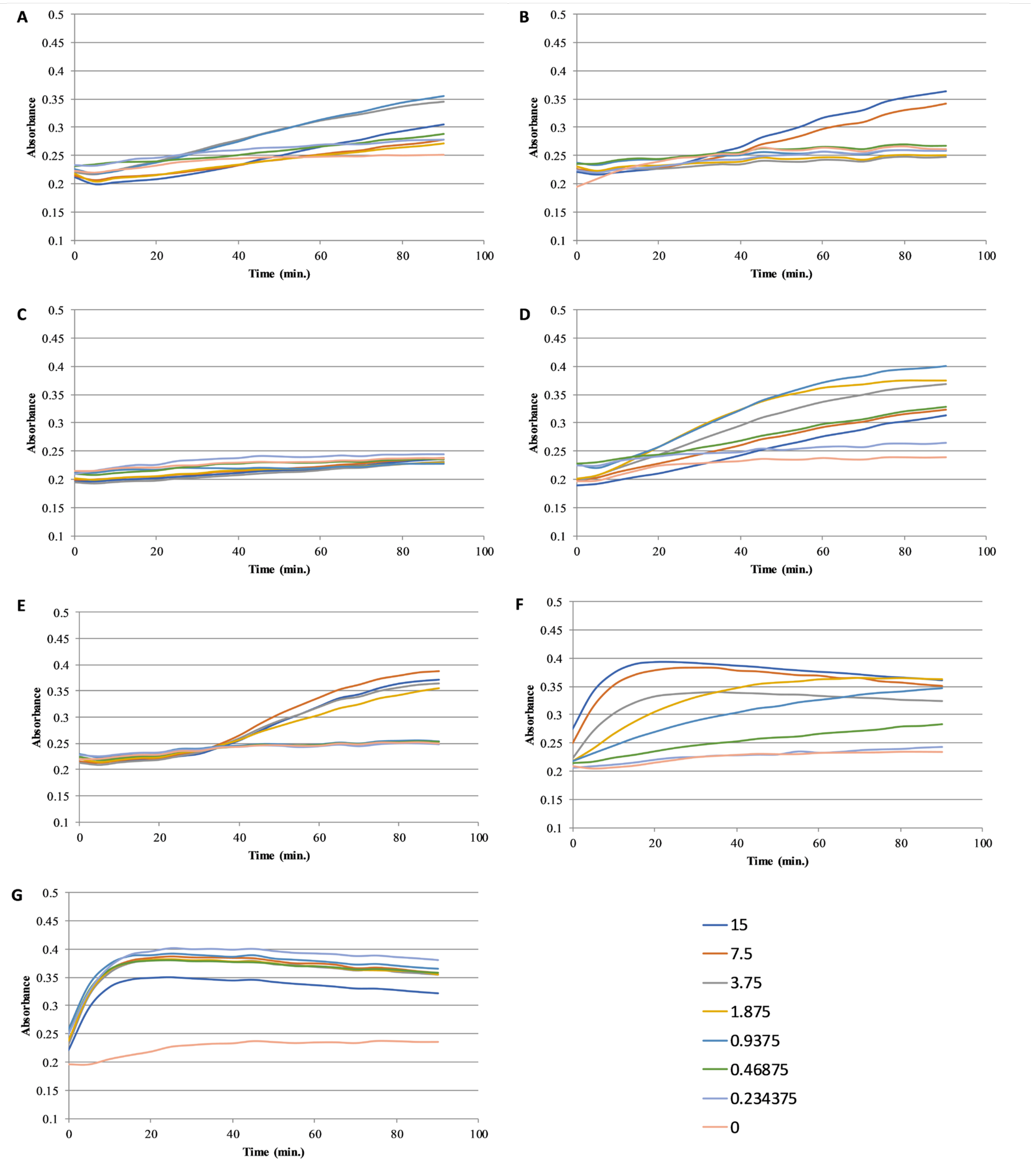

Supplemental Figure S2 – *E. coli* outer membrane permeabilization. The hydrolysis of nitrocefin by β–lactamase was monitored through the evolution of absorbance at 486 nm. Measurements were recorded in 5 minute increments. The peptide concentration for each curve is color coded and noted in the bottom right of the figure in μM units for the to (A) L1, (B) L1V, (C) L1I, (D) L1L, (E) L1A, (F) L1F, (G) Polymyxin B Sulfate (positive control). Data shown are the average of 3 samples.

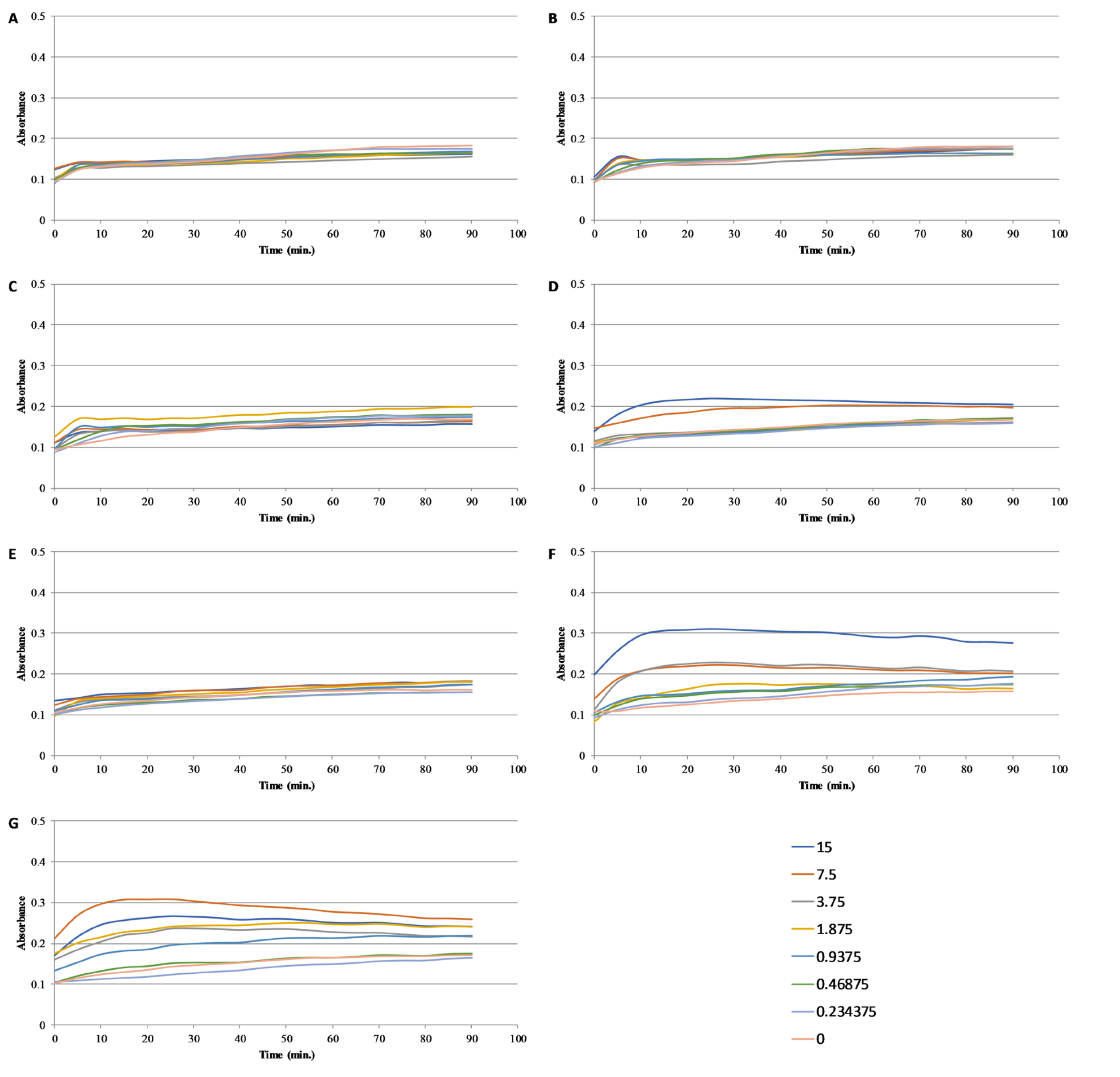

Supplemental Figure S3 – *E. coli* inner membrane permeabilization. The hydrolysis of ONPG by β–galactosidase was monitored through the evolution of absorbance at 420 nm. Measurements were recorded in 5 minute increments. The peptide concentration for each curve is color coded and noted in the bottom right of the figure in μM units for the to (A) L1, (B) L1V, (C) L1I, (D) L1L, (E) L1A, (F) L1F, (G) CTAB (positive control). Data shown are the average of 3 samples.

a	Structure predicition	% Helix	% Sheet
L1	CHHHHHHHHCCCHHHHHHHHHCCC	70.83%	0.00%
L1V	CHHHHHHHHCCCCCSSSSSSSSCC	33.33%	33.33%
L1L	CHHHHHHHHHCCHHHHHHHHHCCC	75.00%	0.00%
L1F	CHHHHHHHHCCCHHHHHHHHCCCC	66.67%	0.00%
L1A	HHHHHHHHCCCCHHHCCCCCCCCC	45.83%	0.00%
L1I	CHHHHHHHCCCCCHHHHSSHSSCC	50.00%	16.67%

b

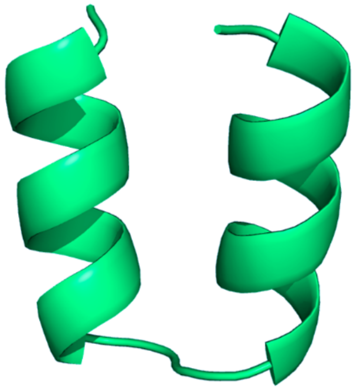

c

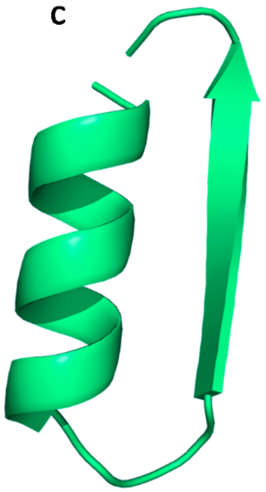

Supplemental figure S4 – Structure prediction of peptides by the iTasser algorithm. (a) Table of amino acid structural predictions in each peptide with total percent helix and sheet calculated. (b) 3D model of the predicted structure of L1 in solution. (b) 3D model of the predicted structure of L1V in solution. All predictions were calculated by the iTasser algorithm (https://zhanglab.ccmb.med.umich.edu/I-TASSER/)

Supplemental Table 1: TCE quenching (Ksv [M^{-1}])

Peptide	
L1V	9.47 ± 1.29
L1I	7.59 ± 4.9
L1L	7.39 ± 3.47
L1A	4.01 ± 0.91
L1F	8.08 ± 5.0
